# Factors associated with depressive symptoms among returnee migrants and non-migrants working adults in Madi municipality in Nepal: a community-based cross-sectional study

**DOI:** 10.1186/s12889-024-18313-3

**Published:** 2024-03-20

**Authors:** Pratik Adhikary, Hridaya Raj Devkota, Arthur L. Reingold, Dirgha J. Ghimire

**Affiliations:** 1 School of Public Health, UC Berkeley, Berkeley, USA; 2 Institute for Social and Environmental Research (ISER-N), Bharatpur, Nepal; 3 Community Support Association of Nepal (COSAN), Kathmandu, Nepal

**Keywords:** Mental health, Working population, Labour, Migration, Depressive symptoms

## Abstract

**Background:**

Mental health is a growing concern worldwide. It is not well understood whether international labour migrants from Nepal who return to Nepal are at higher risk of developing mental health problems. The purpose of our study was to determine the prevalence of and examine the associated factors for depressive symptoms among returnee migrants and non-migrant working male adults in Nepal.

**Methods:**

A cross-sectional survey of a probability-based sample of 725 participants was conducted in February 2020. The sample was comprised of two groups based on migration status: returning migrants and non-migrants. The 21-item Beck Depression Inventory (BDI-21) questionnaire was used to assess depressive symptoms. Logistic regression was applied to investigate factors associated with symptoms of depression.

**Results:**

The overall prevalence of depressive symptoms was 10.1%. However, the prevalence of depressive symptoms was lower (7%) among returnee migrants compared to non-migrants (13.7%). Men in the lower income group had a higher chance of having depressive (AOR = 5.88, 95% CI: 2.17–15.96) than those in the higher income group. Similarly, Buddhists and Christians were more likely to be depressed (AOR = 2.20, 95% CI: 1.03–4.68) than Hindus. Participants with more than two children had a higher chance of having of depressive symptoms (AOR = 4.80, 95% CI: 1.15–20.05) compared with those without children. Unmarried men were more likely to be depressed (AOR = 4.07, 95%, CI:1.11–14.92) than those who were married.

**Conclusion:**

The working Nepali adult male population in Nepal, including returning migrants, is at risk of depressive symptoms, but this association was lower in those in the higher income group, returnee migrants, those who were married, Hindus and those with no children. Our results highlight the need to monitor and develop national policies to ensure the mental health of the Nepali male adult population, including returnee migrants.

## Introduction

An estimated 350 million people worldwide suffer from depression [1]. Although the prevalence of depression varies considerably within and between countries [2, 3], more than two thirds (70%) of the global mental health burden occurs in Low and Middle Income Countries (LMICs) [4]. Mental health problems, including depression, are common in Nepal and a key contributor to morbidity [5, 6]. Kecskes (2015) found that depression is highly prevalent in Nepal, accounting for the second highest rate of depression-related ‘‘disability adjusted life years’’ in the world [7].

Previous studies have shown a relationship between depression and work, for example, workers who report lack of decision latitude, job strain, job insecurity, long working hours and bullying, will experience increasing depressive symptoms [8, 9]. One review found that those working in male-dominated industries are at higher association of depression than the general population [10]. Low income is a commonly reported associated factor for depression [11–13]. Also, people working in another country, so called migrant workers, experience many migration-related stressors that affect their mental health [14, 15]. The social capital theory of migration asserts that an individual’s social networks significantly shape their migration decision and experiences [16, 17]. Social capital, encompassing trust, reciprocity, information, and support, aids migrants in overcoming challenges and adapting to new environments. People are drawn to migrate where existing connections exist, offering practical assistance and insights [18]. Fresh networks in destination countries aid integration and resource access. This theory also reveals positives like job access, but negatives like exploitation or isolation within networks. Essentially, it highlights social connections’ role in migration choices and outcomes, providing a framework for understanding migrants’ challenges, success, and well-being in new settings [19, 20]. Workplace environment, individual factors and socio-economic status all influence the mental health of workers [21–24]. A recent mental health study in Nepal found that 3.4% of adults experience a depressive symptoms [5]. An explorative study on Nepali adults (both migrant and non-migrant workers) revealed that they experienced poor mental health due to adverse living and working conditions, unmet familial and financial needs and unhealthy lifestyles [25].

The overwhelming majority of labour migrants from Nepal are male. The most popular labour destinations for male Nepali migrant workers are the Gulf countries and Malaysia [26]. The evidence highlights that Asian migrants in the Middle-East are at high risks of mental illness due to the living and working conditions [27]. Other evidence suggests that Nepali male migrants in the Middle Eastern countries experience mental health issues, headache and suicide attempts [28]. A better understanding of the prevalence of and associated factors for depressive symptoms among working men (both migrants and non-migrants) could inform the development of policies and tailored workplace mental health interventions. The associated factors for depressive symptoms among the adult male population (both migrants and non-migrants) of Nepal have, however, only been the subject of a relatively small amount of research to date. This cross sectional survey was undertaken to examine the associated factors for depressive symptoms among male adults in Nepal.

## Methods

### Study design and sample

This study was part of a larger project entitled ‘‘Prevalence of depressive symptoms, anxiety and alcohol use disorders among out-migrant labour in Madi Chitwan’’. The current paper is based on the quantitative study which collected data from 725 respondents (with 372 migrants and 353 non-migrants).

A cross-sectional study using multi-stage sampling was conducted among adult male Nepali workers including non-migrants and returnee migrants. The study was conducted in Madi Municipality of Chitwan district, which is located in southern Nepal. The study site was chosen based on the high prevalence of labour out migration [29]. Returning migrants were defined as Nepali citizens ≥ 18 years of age who had worked outside Nepal, mainly in the Middle East, South East Asia and other Asian countries, for more than six months and who had returned to Nepal. Migrants who had returned up to two years previously were included in this study. Returning migrants who had worked in India, Europe, Canada or Australia were excluded from the study. The key reason for exclusion of migrants who returned from Europe, Canada and Australia was their high educational level, high income and higher skillful jobs [30]. The work environment and risk perceptions of workers who worked in developed countries might be different from those individuals who worked in the Gulf, South East Asia and other Asian countries [31]. Similarly, migrants who had worked in India were excluded from this study due to the seasonal nature of migration and the difficulty of identifying them, as well as the fact that their characteristics and risk perceptions are likely to differ from those of migrants who were enrolled in our study. Non-migrants were defined as Nepali citizens ≥ 18 years of age, working in Nepal who had never migrated abroad for work. Non-migrants who had come from other district but working and living temporarily in study sites (district) were excluded from this study. The sample size was determined based on a prevalence of depression of 20.0% among migrant workers in Saudi Arabia, with a 5% margin of error, and the confidence interval was set at 95% [32]. After adjusting for a non-response rate of 20%, the total sample size was 725.

### Sampling process

We used a multi-stage sampling method to select a representative sample from the study population. In the first stage, Madi Municipality of Chitwan district was purposively selected based on its high rate of out-migration for work. As no official records of returnee migrants in the municipality or at the ward level were available, we undertook a mapping exercise to estimate the number of returnee migrants. In the second stage, four wards of Madi Municipality were randomly selected for household listing. Out of 4463 households, male workers from 1743 households had migrated for work abroad and male workers from 2720 households had never migrated. Male workers were categorized into two groups: (a) migrants (returnee migrants) and (b) non migrants (never migrated) to determine the required sample size for each sub group. Although individuals from 1743 households had migrated for work abroad, households listing identified that very limited migrants were returned home during study period. Hence, a decision was made to include those migrants who had returned up to two years previously. Finally, all returnee migrants who met eligible criteria were selected for study, while non-migrants were randomly selected by a computer-based random selection technique.

### Survey instruments

A previously validated questionnaire [33] was used to collect data on socio-demographic, mental health-related measures, and other characteristics of the participants. We selected eleven variables for analysis, although our questionnaire included 18 questions related to personal characteristics. The eleven variables selected for inclusion in the multivariate analysis were age (classified into three different groups), ethnicity, religion, education, marital status, number of children, income, area of land, household asset index, migration status and health insurance. The focus of this study was on a single outcome variable: having had an experience of depressive symptoms in last two weeks. The 21-item Beck Depression Inventory (BDI-21) questionnaire was used to assess depressive symptoms [34] and the tool was observer rated instead of self-reported. In Nepal, the BDI-21 has been validated for detection of depressive symptoms in the primary healthcare setting [33]. A pilot study was conducted with ten workers (five returnee migrants and five non-migrants) in a similar locality, and questions and words found to be difficult to understand were modified.

### Data collection procedures

Twelve experienced data enumerators and three supervisors in quantitative data collection were recruited and trained. The data enumerators had at least a bachelors’ degree qualification in social science and health education. Preference was given to local candidates from the survey districts who were familiar with the local context and who spoke local languages. Three days of training was provided to the data enumerators on the purpose of the study, study tools and methods, ethical matters and the recruitment process. The questionnaire was administered using pen-and-paper personal interviews (PAPI). Survey data were collected in January and February, 2020. Field supervisors were responsible for overseeing participant recruitment, data collection and checking, and checking data quality. The entire data-collection process was closely monitored by the supervisors.

### Data cleaning and management

The data enumerators spot-checked completed survey questionnaire data to minimize errors and missing information. Data were also checked for completeness and consistency by the field supervisors during data collection, and survey data were entered into EXCEL software by trained and experienced data entry clerks. All data were entered twice to check the quality of the data-entry process [35].

### Data analysis

Statistical Packages for the Social Sciences (SPSS version 22) was used for data analysis. Frequencies and percentages were used to summarize the characteristics of the participants. The data from the BDI-21 questionnaire were analysed using a dichotomous score for each of the 21 questions. Participants with an overall score of > 16 were defined as having a depressive symptoms and those with a score of ≤ 16 were defined as having no depressive symptoms [33]. In other words, the dependent variable was: depressive symptoms (0 = no depression and 1 = depression). The independent variables were categorical. The household assets index variable was constructed using 12 indicators of household possessions having a total score of 12. Household possession items were chosen from the list of The Nepal Demographic Health Survey (NDHS) 2016. All the items were weighted equally and made into dichotomous variables assigning the code 0 or 1, with higher values reflecting more asset ownership. The score was divided into quintiles for the purpose of analysis; the lowest two and the highest two groups were collapsed to make three categories (low, middle and high income) of index ranking. Multivariable logistic regression models were fitted to assess the association of the outcome variable (depressive symptoms) with various associated risk factors. Odds ratios were used to determine the strength of association for selected variables, with a cut off for statistical significance set at *p* = 0.05.

## Results

### Socio-demographic characteristics of participants

The total number of workers who participated in this study was 725, of whom, 372 (51.3%) were migrants (returnee migrants) and 353 (48.7%) were non-migrants. The response rate was 96%. Only 29 workers refused to take part in this study, due to poor timing (work on their farms). The mean age of the study participants was 37.5 years, ranging from 18 to 59 years (SD = 9.63). The vast majority (87.3%) of participants were Hindu and married (91.4%), and over half (56.8%) had 1–2 children and had a secondary/SLC level of education (58.5%) (Table 1). In terms of caste ethnicity, (43.4%) were Brahmin/Chhetri/Thakuri. Two thirds (65.5%) were semi-skilled or unskilled workers and the majority (69.2%) worked more than 50 h per week. Just over half (53.1%) had health insurance, while almost half (44.3%) had an income of less than $267 (NRs 30,000) per month.

**Table 1:** Characteristics of study participants (*n* = 725)

Variable	Migrants (n) %	Non-migrants (n) %	Total (n) %
**Depression**			
No	346 (93.0)	306 (86.7)	652 (89.9)
Yes	26 (7.0)	47 (13.3)	73 (10.1)
**Age**			
< 30 years	109 (29.3)	59 (16.7)	168 (23.2)
30–39 years	154 (41.4)	93 (26.3)	247 (34.1)
≥ 40 years	109 (29.3)	201 (56.9)	310 (42.8)
**Ethnicity**			
Brahmin/Chhetri/Thakuri	165 (44.4)	150 (42.5)	315 (43.4)
Dalit	53 (14.2)	48 (13.6)	101 (13.9)
Janajati/Madheshi	154 (41.4)	155 (43.9)	309 (42.6)
**Religion**			
Hindu	334 (89.8)	308 (87.3)	642 (88.6)
Baudha/Christian	38 (10.2)	45 (12.7)	83 (11.4)
**Marital Status**			
Married	342 (91.9)	321 (90.9)	663 (91.4)
Unmarried	30 (8.1)	32 (9.1)	62 (8.6)
**Children**			
No Children	65 (17.5)	47 (13.3)	112 (15.4)
1–2 Children	244 (65.6)	168 (47.6)	412 (56.8)
>Two Children	63 (16.9)	138 (39.1)	201 (27.7)
**Education**			
Higher Education	36 (9.7)	52 (14.7)	88 (12.1)
Secondary/SLC	260 (69.9)	164 (46.5)	424 (58.5)
No Edu./Primary	76 (20.4)	137 (38.8)	213 (29.4)
**Occupation**			
Service/Business	8 (2.2)	147 (41.6)	155 (21.4)
Agriculture	3 (0.8)	92 (26.1)	95 (13.1)
Semi-skilled Worker	280 (75.3)	32 (9.1)	312 (43.0)
Unskilled Worker	81 (21.8)	82 (23.2)	163 (22.5)
**Income in Nepalese rupees (per month)**			
> 40,000 ($356)	242 (65.1)	23 (6.5)	265 (36.6)
30,000–40,000 ($267-$356)	102 (27.4)	37 (10.5)	139 (19.2)
< 30,000 ($267)	28 (7.5)	293 (83.0)	321 (44.3)
**Wealth Index**			
Rich	101 (27.2)	93 (26.3)	194 (26.8)
Middle	211 (56.7)	155 (43.9)	366 (50.5)
Poor	60 (16.1)	105 (29.7)	165 (22.8)
**Health insurance**			
**Yes**	227 (61.0)	158 (44.8)	385 (53.1)
**No**	145 (39.0)	195 (55.2)	340 (46.9 )

### Prevalence and severity of depressive symptoms

A noteworthy percentage of individuals who did not migrate (13.2%) exhibited signs of depressive symptoms, whereas 7% of migrants reported experiencing such symptoms. Among the non-migrant group, a larger proportion (7.6%) displayed mild symptoms, 4.8% showed moderate symptoms, and slightly less than 1% presented severe levels of depressive symptoms (Table 2).

**Table 2:** Prevalence of depressive symptoms

Severity level (Score)	Depressive Symptoms					
Depression	Migrants		Non-migrants		Total	
	*n* = 372	%	*n* = 353	%	*n* = 725	%
Minimal (0–16)	346	93.0	306	86.7	652	89.9
Mild (17–20)	16	4.3	27	7.6	43	5.9
Moderate (21–30)	7	1.9	17	4.8	24	3.3
Severe (31–63)	3	0.8	3	0.8	6	0.8

### Factors associated with depressive symptoms among male adults

In the univariate analysis, migration status, wealth index, income, occupation, number of children, education, religion and health insurance were significantly associated with depressive symptoms (p-values 0.005).

Multivariable logistic regression analysis was used to adjust for all factors and to find significant associations with depression status. The findings of the multivariable logistic regression analysis showed that men in the lowest income group [<$267 (NRs. <30,000] and those with > 2 children had six (AOR = 5.88, 95% CI:2.17–15.96) and five (AOR = 4.80, 95% CI: 1.15–20.05) times higher odds of having depressive symptoms compared to those of highest income group and with no children respectively. Similarly, unmarried men and men of the Buddhist and Christian faith had four (AOR = 4.07, 95% CI: 1.11–14.92) and two (AOR = 2.20, 95% CI: 1.03–4.68) higher odds than married and Hindu men, respectively. However, migration status was not statistically significant when adjusted (Table 3).

**Table 3:** Predictors of depression among 725 returnee migrants and non-migrants working adults in Nepal

Variable	Depression			
	OR (95% CI) Unadjusted	p-value	AOR (95% CI) Adjusted	p-value
**Demographic and Socio-economic characteristics**				
**Migration status**				
Migrants	1.0		1.0	
Non-migrants	0.49 (0.30–0.81)	**0.005**	2.25 (0.96–5.29)	0.062
**Age**				
<30 years	1.0		1.0	
30–39 years	1.17 (0.57–2.38)	0.674	1.15 (0.49–2.73)	0.749
>40 years	1.67 (0.86–3.22)	0.130	1.07 (0.42–2.69)	0.893
**Ethnicity**				
Brahmin/Chhetri/Thakuri	1.0		1.0	
Dalit	1.86 (0.95–3.66)	0.072	1.01 (0.46–2.21)	0.990
Janajati/Madheshi	1.19 (0.69–2.04)	0.530	0.66 (0.33–1.32)	0.244
**Religion**				
Hindu	1.0		1.0	
Buddha/Christian	2.01 (1.06–3.78)	**0.031**	2.20 (1.03–4.68)	**0.041**
**Education**				
Higher Education	1.0		1.0	
Secondary/SLC	0.88 (0.37–2.08)	0.772	0.75 (0.29–1.91)	0.546
No Edu./Primary	2.35 (1.01–5.51)	**0.049**	1.19 (0.41–3.38)	0.748
**Marital status**				
Married	1.0		1.0	
Unmarried	1.36 (0.62–2.99)	**0.440**	4.07 (1.11–14.92)	**0.034**
**Health insurance**				
Yes	1.0		1.0	
No	2.08 (1.26–3.42)	**0.004**	1.24 (0.68–2.26)	0.476
**Number of children**				
No Children	1.0		1.0	
1–2 Children	1.35 (0.58–3.13)	0.486	3.28 (0.84–12.91)	0.089
> 2 Children	2.84 (1.21–6.67)	**0.016**	4.80 (1.15–20.05)	**0.032**
**Income**				
**>**40,000 ($356)	1.0		1.0	
30,000–40,000 ($267-$356)	1.98 (0.84–4.70)	0.119	1.99 (0.81–4.84)	0.132
**<**30,000 ($267)	4.36 (2.22–8.56)	**< 0.001**	5.88 (2.17–15.96)	**< 0.001**
**Wealth Index**				
Rich	1.0		1.0	
Middle	1.33 (0.68–2.61)	0.399	0.89 (0.43–1.86)	0.760
Poor	2.85 (1.42–5.70)	**0.003**	1.27 (0.52–3.06)	0.601

Note: BDI cut of score 16:17 (or 17 or more) is considered to indicate depression. [Score 0–16 = No depression, 17 through highest = Depression]; B/C/T = Brahmin/Chhetri/Thakuri

## Discussion

The overall prevalence of depressive symptoms among participants in this study was 10.1%; it was, higher (13.7%) among non-migrants than among returnee migrants (7%). Due to a lack of prior studies on the prevalence of depressive symptoms in Nepal using the BDI, our findings can only be compared with those of studies using different survey tools in Nepal and other countries. Furthermore, many earlier studies focused solely on mental health disorders, and these studies often used a range of mental-health related questions and scales.

The prevalence of depressive symptoms we found (10.1%) is comparable to that reported in a cross sectional study among Nepalese adults aged 18–65 years (11.7%) [6] and studies of non-Nepalese populations, including those in Malaysia (10.3%) [36], and the United States (16%) [37]. However, the prevalence of depressive symptoms we found was moderately higher than that in the study recently conducted by Devkota et al., (2020) (7%) in Nepal [38] and in the study by Haeffner and Santana (2019) (6.2%) in Brazil [39] and about three fold higher than the national prevalence (2.7%) in India [40]; a recent pilot study for a national mental health survey in Nepal reported on estimated national prevalence of 3.2% major depressive disorder in Nepal [5]. One possible explanation as to why the prevalence of depressive disorder found in various studies is the use of different instruments to measure mental health and the use of different cut points in the analyses. In our study, two likely explanations for the higher prevalence of depressive symptoms among non-migrants are the higher income and savings of returnee migrants, as well as the fact that they returned home with money and savings and in a good mood because they were reunited with family and friends. Similarly, a third possible explanation for higher prevalence of depressive symptoms among non-migrants could also be the influence of a difference in the lifestyle in these two locations. This would suggest that non-migrants in Nepal has lower health and safety standards and limited entertainment, but there is no available published evidence to back up this assertion.

In the multivariable analysis, depressive symptom was associated with a lower level of income, with returnee migrants who earned <$267 (NRs. <30,000) per month being more likely to have depressive symptoms than those who earned >$356 (> NRs. 40,000) per month. Psychological distress, including depression, has been associated with low income in prior studies [41, 42]. A low income is a commonly reported associated risk factor for depression [11–13, 43, 44], although one study of Cambodian migrants in Thailand found that those with a higher income were more likely to experience depression [45]. One plausible reason for depressive symptoms in migrants with a higher income is that migrant workers had jobs with long working hours and without a break, and so had less time for rest, leisure and social relationships [11].

In our study, although having more children was not associated with depressive symptoms. Participants (both returnee migrants and non-migrants) who had more than two children were more likely to meet criteria for depressive symptoms, compared to those participants who had no children. In contrast, Donato et al. (2020) found that having more children in the household was associated with a slightly lower risk of depression [46]. According to our study, one potential explanation for this is that participants with more children are more stressed due to the fact that they are under more demands from their family and children, as well as the possibility that they feel more responsible for their children and other dependents.

We also found an association between marital status and depression such that unmarried men were more likely to have depression compared to married men. This finding differs from that of a previously published report [47].

We also found a significant relationship between religion and depressive symptoms, a higher rate of depressive symptoms among participants from Buddhist or Christian religious backgrounds compared to those from Hindu background. However, Xu and colleagues (2020) found that Buddhist respondents reported lower levels of depressive symptoms compared to non-Buddhist counterparts in a study of married women in Thailand [48]. Possible reasons for a higher prevalence of depressive symptoms among Buddhist or Christian religious group in our study could include a lower religious involvement and practicing mantra and meditation [49]. Religion could play an important role in many situations, as religious beliefs and practices influence mental health and wellbeing [50–52] as documented in prior studies.

Our findings closely align with network and social capital theory. According to this hypothesis, interpersonal connections between current migrants, former migrants, and non-migrants to both the place of origin and destination cause migration to occur [16, 17]. These inter-personal connections or networks among migrants help to promote employment opportunities in the country of destination while also lowering the costs and hazards of migration. Exchanges, duties, and shared identities that result from social networks and linkages give each person access to resources and the possibility of support [18]. Migrants may have the most antisocial and bad experiences including mental illness, unemployment and family conflicts in their host country if there are no shared rights, obligations, and social networks [19, 20]. The Social Capital hypothesis may be useful in detecting probable mental health issues in Nepalese migrant workers.

Our study provides an in-depth examination of depressive symptoms among returnee migrants and non-migrant working male adults in Nepal. While the government of Nepal has established a national mental health policy, it is evident that there is a need to enhance the integration of mental health care within the primary health care system. A large portion of mental health services currently relies on secondary, tertiary, or specialized care, along with private and NGO-led initiatives, which are not always accessible to the wider population.

Given the limited government allocation to mental health, which is less than 1% of the total health budget, our study underlines the importance of strategic planning and investment to broaden access to mental health resources. We recommend a reassessment of the current mental health funding with a view of expanding service accessibility at the community level, particularly for those in the lower-income bracket, as socio-economic status was found to be a significant factor in depressive symptoms.

Enhanced public health strategies can be aimed at increasing the awareness and destigmatization of mental health issues through targeted campaigns and incorporating mental health discussions in educational settings. By doing so, we can hope to address some of the disparities in mental health service utilization and outcomes among different demographic groups within the Nepali male adult population.

### Strengths and limitations

This study represents one of the first community-based studies of the mental health-migration relationship comparing returned migrants and non-migrants in Nepal. Hence, this study adds to the literature on mental health inequalities among migrants and non-migrants in low and middle-income countries. Strengths of our study include adequate sample size (*n* = 725). Moreover, this study used validated tools to measure the mental health outcome. However, our study also had several limitations. First, this was a cross-sectional study and it is impossible to establish cause-effect relationships between depressive symptoms and the various socio-economic factors. Second, this study was carried out only in Madi municipality of Chitwan district, and the findings may not be generalizable to other settings in Nepal. Third, our study population was composed only of men as there were no female returnee during our study period, and our findings are generalizable only to the male populations of selected migrant-sending communities in Nepal. Fourth, potential limitation of this study is the non-adjustment or lack of a weighting procedure in the statistical analysis of the complex sample survey, which may impact the precision of our estimates, including odds ratios (OR) and their corresponding 95% confidence intervals (CI 95%). Similarly, this study excluded returnee migrants from high income countries (or those group of returnee migrants with high level of education, income and employment) and India as there was no returnee migrants from high income countries and returnee migrants from India were seasonal migrants (worked less than six months in India) and we only included returnee migrants who had at least six-month work experience abroad. Further, there may be differences in the ways in which returned migrants and non-migrants perceive their mental health.

## Conclusion

This study has identified socio-economic status, religion, marital status, and the number of children as important factors associated with depressive symptoms among working Nepali adult males, including both returnee migrants and non-migrants. The findings suggest that returnee migrants have a lower prevalence of depressive symptoms compared to non-migrants, highlighting the nuanced nature of mental health issues within this population.

As the World Health Assembly resolution 61.17 calls for the enhancement of migrants’ well-being, our research emphasizes that efforts should also focus on the broader adult working population in Nepal. There is a clear need for the effective implementation and active enforcement of mental health policies that consider the diversity within the population.

To address these issues comprehensively, we advocate for the reform of health promotion programs to include a holistic approach to mental health advocacy. This should involve not only the recruitment of additional mental health specialists who can provide care and guidance but also a systemic integration of mental health awareness into public campaigns and educational curricula. These initiatives are vital to foster a society where both migrants and non-migrants receive the mental health support they need and where well-being becomes an achievable goal for every individual engaged in the labor force of Nepal.

## Data Availability

The datasets used for this study are available from the corresponding author on reasonable request.

## Abbreviations

BDI: Beck Depression Inventory
IOM: International Organization for Migration
ISERN: Institute for Social and Environmental Research
LMICs: Low and Middle Income Country
NDHS: Nepal Demographic Health Survey
NHRC: Nepal Health Research Council
PAPI: Pen-and-Paper Personal Interviews
SPSS: Statistical Packages for the Social Sciences
WHA: World Health Assembly
WHO: World Health Organization
